# Long-Term Monitoring of Radioactive Cesium in Commercial Foods and Dietary Intake Estimated by Total Diet Studies in Japan after the Fukushima Daiichi Nuclear Power Plant Accident

**DOI:** 10.14252/foodsafetyfscj.D-26-00006

**Published:** 2026-09-25

**Authors:** Hiromi Nabeshi, Tomoaki Tsutsumi

**Affiliations:** Division of Foods, National Institute of Health Sciences, 3-25-26, Tonomachi, Kawasaki-ku, Kawasaki, Kanagawa 210-9501, Japan

**Keywords:** radioactive cesium (^134^> Cs and ^137^> Cs), strontium-90, Fukushima Daiichi Nuclear Power Plant accident, total diet study, food monitoring

## Abstract

Following the Fukushima Daiichi Nuclear Power Plant accident in 2011, Japan implemented extensive monitoring of radioactive materials in foods and established strict regulatory limits based on an annual effective dose of 1 mSv/year from artificial radionuclides. This review summarizes long-term surveillance data on radioactive cesium (r-Cs) in commercial foods and long-term assessments of dietary intake of r-Cs and strontium-90 (^90^Sr) using the total diet study (TDS)/market basket (MB) method. Monitoring of commercial foods demonstrated that pre-shipment inspections and production controls are highly effective for cultivated agricultural products and livestock products, with regulatory exceedances occurring almost exclusively in wild mushrooms, wild edible plants, and wildlife-derived foods. No r-Cs has been detected in any infant food sample since monitoring began. TDS-based assessments show that the committed effective dose from r-Cs declined rapidly during the first few years after the accident and has remained at a very low level (generally below 0.001 mSv/year) nationwide since around 2017, while ^90^Sr levels exhibited no clear regional differences and remained comparable to pre-accident background levels. These findings confirm that Japan’s long-term food safety controls have kept internal radiation exposure far below international safety limits. The integration of surveillance, TDS monitoring, and risk management provides a robust evidence-based model for ensuring food safety following nuclear events.

## 1. Introduction

The accident at the Fukushima Daiichi Nuclear Power Plant (hereafter, the Fukushima accident), triggered by the Great East Japan Earthquake on March 11, 2011, resulted in the release of radioactive materials into the environment and the contamination of food. In response, the Ministry of Health, Labour and Welfare (MHLW) established provisional regulation values for radioactive materials in food only six days after the accident, based on the index values for food intake restrictions recommended by the Nuclear Safety Commission^1^^)^. The provisional values were designed to ensure that the annual dose from food (excluding natural radionuclides) would not exceed 5 mSv/year. Although the Food Safety Commission later confirmed that foods below these values posed no health concern, the standards were reviewed from a longer-term safety perspective. As a result, new regulation values took effect on April 1, 2012, under the Food Sanitation Act^2^^)^, lowering the allowable annual dose from food to 1 mSv/year, consistent with Codex guidelines for intervention exemption. In this review, the regulatory concentration limits for radioactive cesium established under the Food Sanitation Act (e.g., 100 Bq/kg for general foods, 50 Bq/kg for infant foods) are collectively referred to as the Japanese Maximum Limits (JMLs).

Among the radionuclides released by the Fukushima accident, several were short-lived, such as iodine and tellurium isotopes, and their concentrations declined rapidly within months. One year after the accident, long-lived radionuclides—mainly radioactive cesium (r-Cs: ^134^Cs and ^137^Cs), along with smaller quantities of strontium-90 (^90^Sr) and plutonium—became the primary nuclides of concern in food. Because many radionuclides released in small amounts do not emit gamma rays and are difficult to measure in routine monitoring, the regulatory framework adopted the concentration of r-Cs (sum of ^134^Cs and ^137^Cs) as a practical indicator, taking into account contributions from other radionuclides, such as ^90^Sr, plutonium isotopes, and ruthenium-106, which are estimated to contribute approximately 12% of the total dose for adults^3^^)^. The regulation of food based on r-Cs remains in place 15 years after the accident.

To verify the effectiveness of these regulatory measures, two major surveillance systems were established: (1) monitoring of radioactive cesium concentrations in commercial foods, and (2) total diet studies (TDS) using the market basket (MB) method to estimate internal exposure from actual food consumption. Over the years, these programs have produced a substantial body of monitoring data, forming a strong scientific basis for risk assessment.

Immediately after the accident, many countries imposed import restrictions on Japanese foods out of concern for radiological contamination. These included the European Union, the United States, China, South Korea, and others, which required inspection certificates and restricted products from Fukushima and neighboring prefectures^4^^)^. As Japan implemented strict monitoring, transparent data disclosure, and internationally aligned risk management, many of these restrictions were gradually lifted. In 2023, the EU removed all remaining restrictions^5^^)^, followed by the United States and the United Kingdom in 2024^6^^)^. Nevertheless, some countries maintain regional restrictions, and continuous scientific evaluation remains important for strengthening international confidence.

Based on this background, this review summarizes research on radioactive materials in foods conducted after the Fukushima accident. In particular, we describe (1) the actual concentrations of r-Cs in commercial foods and (2) estimates of internal exposure doses of r-Cs and ^90^Sr using the TDS approach. Based on these findings, we discuss the current status and future challenges of food safety management related to radioactive materials.

## 2. Monitoring of Radioactive Cesium in Commercial foods

### 2.1. Survey Design and Analytical Methods

To evaluate the effectiveness of Japan’s regulatory framework and to understand the actual contamination levels of foods available to consumers, nationwide surveys of radioactive cesium in commercial foods have been conducted continuously since fiscal year 2011, when provisional regulation values first came into effect^1^^,^^7^^,^^8^^,^^9^^,^^10^^)^. These surveys targeted general foods (excluding drinking water, milk, and infant foods), and from fiscal year 2012 onward, infant foods were also monitored because of the higher radiosensitivity of infants and the establishment of a stricter JML of 50 Bq/kg^11^^)^.

Sampling was carried out mainly in 17 prefectures designated as priority regions for pre-shipment inspections under government monitoring plans^12^^)^. Based on past findings, surveys increasingly focused on food items with a higher likelihood of containing elevated r-Cs levels, such as wild mushrooms, wild edible plants, and log-grown mushrooms. Food samples were purchased from supermarkets, direct sales outlets, and online stores, and included both fresh and processed products. Infant food samples—such as formula milk, liquid formula, and packaged baby foods—were similarly collected from retail outlets and online markets.

The measurement of r-Cs concentrations followed nationally standardized analytical procedures established by the Ministry of Health, Labour and Welfare^13^^,^^14^^,^^15^^)^. For general foods, samples were first subjected to scintillation-based screening (e.g., NaI(Tl) and CsI(Tl) detectors)^14^^)^; those exceeding the screening threshold (25 Bq/kg) underwent confirmatory γ-ray spectrometry using high-purity germanium (HPGe) detectors^13^^)^. For foods typically consumed after rehydration, concentrations were converted accordingly based on official guidelines^15^^)^. Infant foods were analyzed directly using HPGe detector with extended counting times to achieve detection limits below 5 Bq/kg, or below 1 Bq/kg for drinking water intended for infants. All measured values were corrected for attenuation and decay on the day of purchase.

Through these long-term, standardized surveys, Japan has accumulated a substantial and consistent dataset on r-Cs in commercial foods, forming a robust foundation for assessing contamination trends and verifying the effectiveness of regulatory measures

### 2.2. r-Cs Concentration in General Commercial foods

General food samples with r-Cs concentrations above 25 Bq/kg were defined as “detected samples,” and those exceeding 105 Bq/kg were classified as “samples exceeding the JML,” based on measurement uncertainty requirements specified for HPGe detectors^13^^)^. Because the counting error near the JMLs limit must remain within 10% and results are reported with two significant figures^13^^)^, 105 Bq/kg is used operationally as the threshold for exceedance of the 100 Bq/kg, JML for general foods. Foods were categorized into eight groups: vegetables, wild edible plants, mushrooms, fruits and seeds, cereals and beans, meat and eggs, marine products, and others (including honey, seaweed, and processed mulberry leaves). Processed foods were assigned according to their main ingredient.

Table 1 provides an overview of the number of samples tested, positive detections, and exceedances by food category from FY2012 to FY2019, whileFigure 1 illustrates the annual distribution of tested categories alongside overall detection and exceedance rates. Annual variation in sample numbers and category composition reflects the targeted nature of monitoring, which increasingly focused on food items and regions with higher probabilities of r-Cs detection based on preceding survey findings.

**Table 1.** Summary of Radioactive Cesium Inspections by Food Category (2012–2019)

Food category		Number of samples per year and category							
		2012	2013	2014	2015	2016	2017	2018	2019
Vegetables	Inspection	369	520	516	224	52	201	161	164
	Detection	0	1	1	0	0	0	0	0
	Exceeded JML	0	0	0	0	0	0	0	0
Wild edible plants	Inspection	40	76	90	151	229	185	164	169
	Detection	1	3	7	4	20	19	20	24
	Exceeded JML	0	1	3	0	6	5	6	0
Mushrooms	Inspection	311	296	237	227	258	217	206	196
	Detection	35	34	30	37	37	32	25	17
	Exceeded JML	3	3	6	11	4	4	4	0
Fruits, nuts, seeds	Inspection	322	345	302	192	78	58	86	82
	Detection	7	3	0	3	0	1	0	0
	Exceeded JML	0	0	0	0	0	0	0	0
Grains, beans	Inspection	136	124	95	42	2	32	34	37
	Detection	0	0	0	0	0	0	0	0
	Exceeded JML	0	0	0	0	0	0	0	0
Meat, eggs	Inspection	184	135	72	6	2	22	34	32
	Detection	0	0	0	1	0	0	1	0
	Exceeded JML	0	0	0	1	0	0	0	0
Aquatic products	Inspection	302	173	193	23	13	0	0	2
	Detection	17	7	3	1	0	0	0	0
	Exceeded JML	0	0	0	0	0	0	0	0
Others	Inspection	71	5	11	35	20	0	0	1
	Detection	8	0	0	1	0	0	0	0
	Exceeded JML	0	0	0	0	0	0	0	0
Total	Inspection	1735	1674	1516	900	654	715	685	683
	Detection	68	48	41	47	57	52	46	41
	Exceeded JML	3	4	9	12	10	9	10	5
	Detection rate (%)	3.9	2.9	2.7	5.2	8.7	7.3	6.7	6.0
	Exceeded JML rate (%)	0.17	0.24	0.59	1.3	1.5	1.3	1.5	0.73

JML: Japanese maximum level for radioactive cesium in foods.

**Fig. 1.**
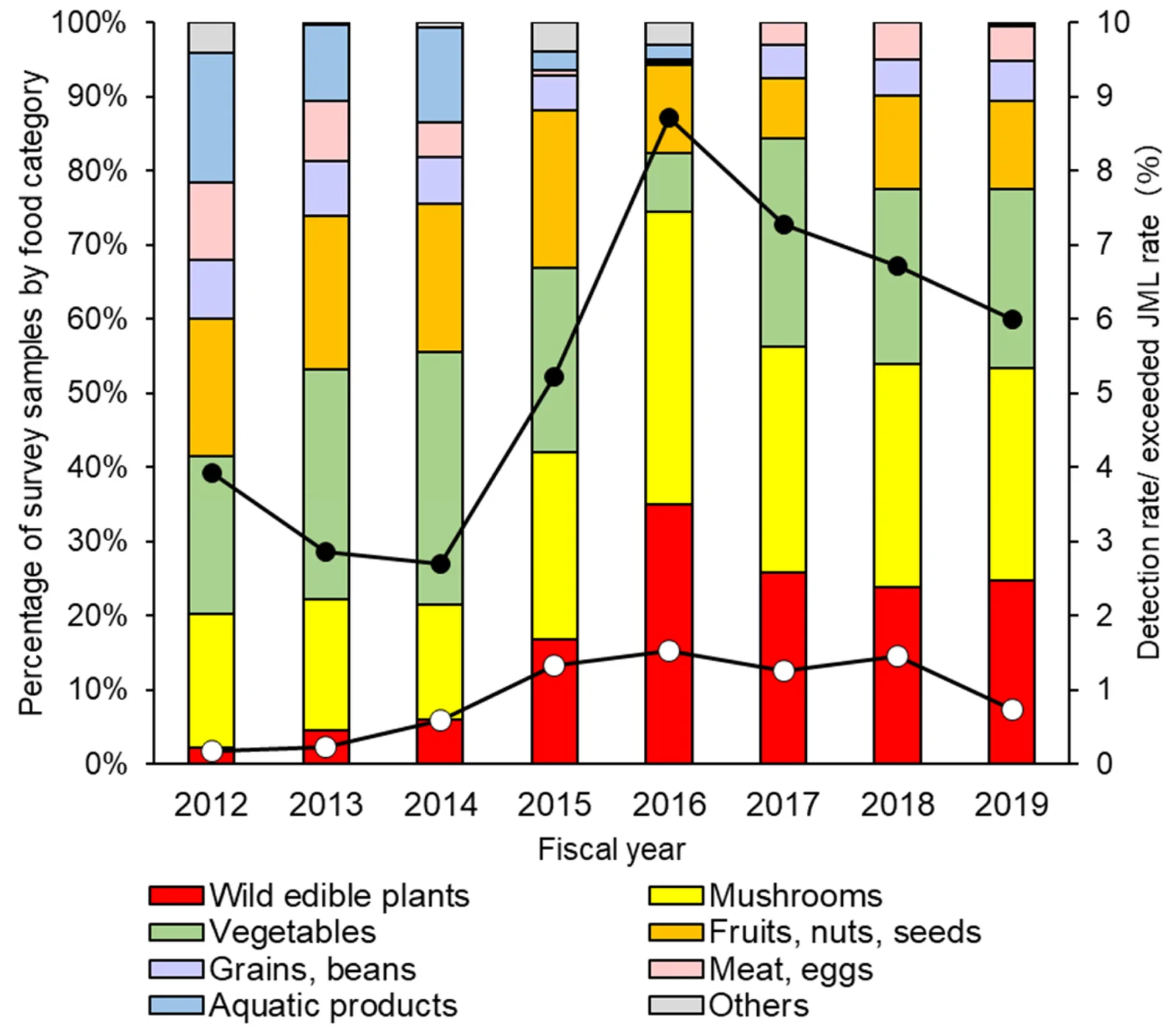
Annual trends in survey proportion by food category, and overall detection and exceedance rate Closed circles indicate detection rates, and open circles indicate exceedance rates.

During FY2012–2014, more than 1,500 samples were collected annually, covering a broad range of foods including vegetables, fruits and seeds, cereals and beans, livestock products, and marine products. In this early period, the detection rate remained below 4%, and the proportion of samples exceeding the JML was below 0.6%, indicating that pre-shipment inspections by local governments effectively prevented the distribution of highly contaminated foods even after the regulatory limit for general foods was substantially lowered^8^^,^^9^^)^. Although r-Cs was detected in several categories—including mushrooms, wild edible plants, fruits and seeds, freshwater fish, and processed mulberry products—only mushrooms and wild edible plants exceeded the JML^8^^,^^9^^)^.

**Fig. 2.**
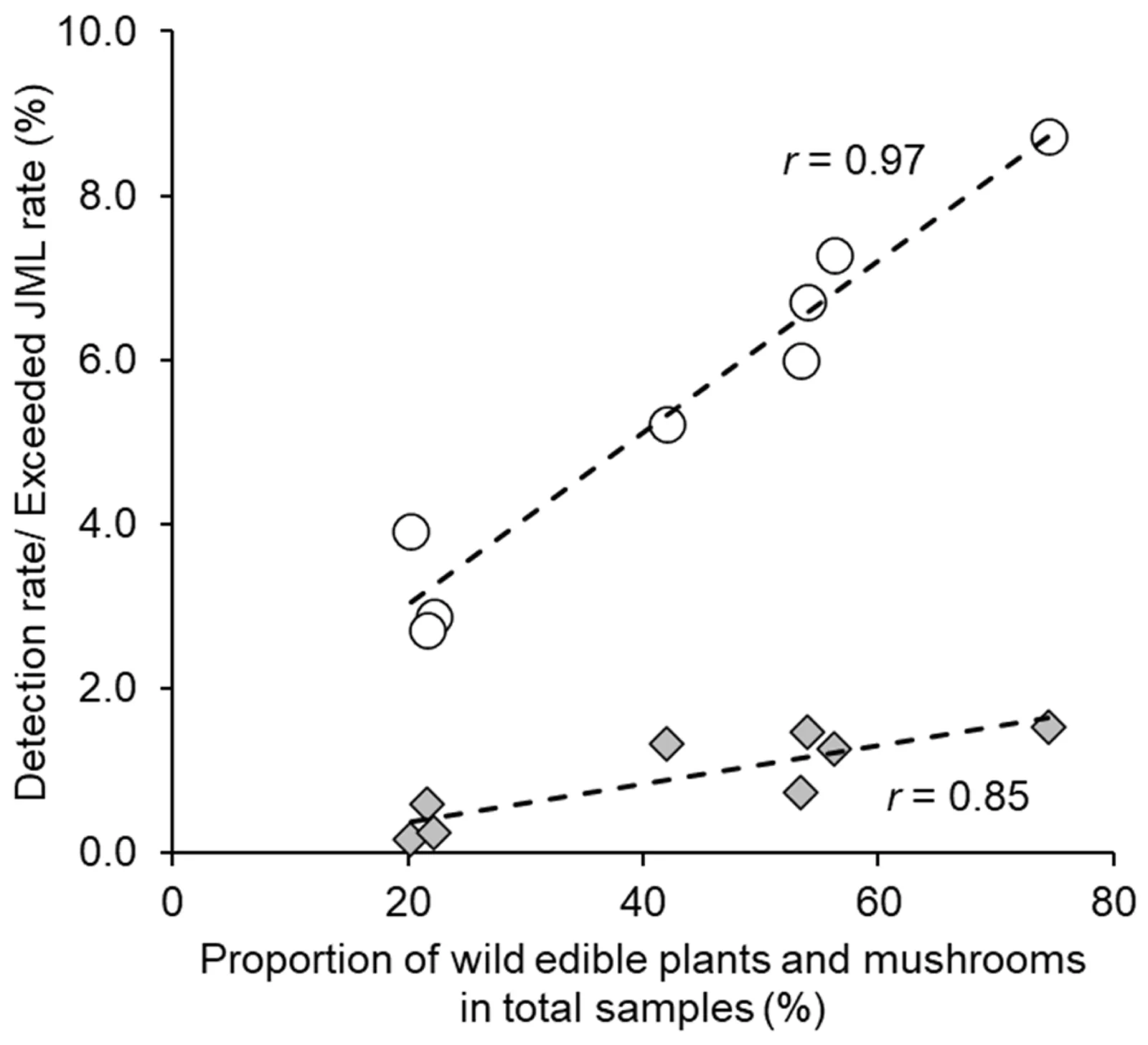
Relationship between detection and exceedance rates and the proportion of wild edible plants and mushrooms in total surveys Open circles indicate detection rates, and closed squares indicate exceedance rates.

Based on these observations, surveys from FY2015 onward were intentionally focused on mushrooms and wild edible plants, which were most likely to contain detectable r-Cs. Monitoring of cultivated vegetables, cereals and beans, livestock products, and marine products was reduced due to their consistently low detection rates. Sampling was expanded from supermarkets in urban regions to direct sales outlets in production regions, and food products from regions with higher detection likelihood were actively purchased. Consequently, the overall detection rate increased to approximately 5–9%, and the proportion exceeding the JML rose to 1–1.5%. Figure 2 shows that both the detection rate and exceedance proportion closely correlated with the proportion of mushrooms and wild edible plants in the annual sample set *(r* = 0.97 for detection rate), indicating that these increases primarily reflect targeted sampling rather than widespread increases in contamination levels. In fact, more than 87% of detected samples and more than 90% of exceedances each year originated from these two categories.

**Fig. 3.**
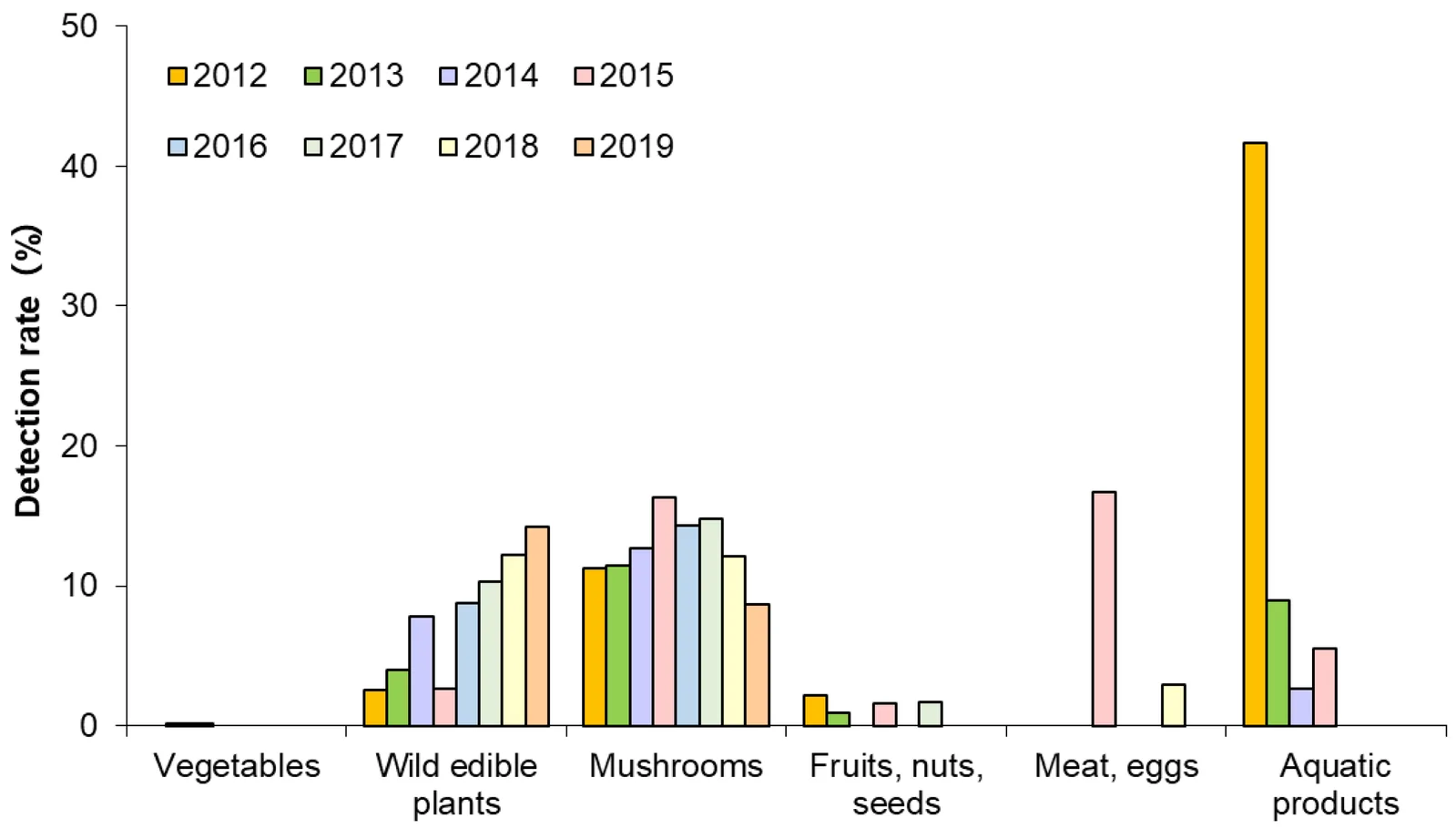
Annual trends in detection rates by food category

The detection rates by individual food category (Fig. 3) show distinct patterns. Freshwater fish exhibited high detection rates immediately after the accident, exceeding 40%, but gradually declined to 0% by FY2016, and only a small number of such samples were tested thereafter. In contrast, detection rates for wild edible plants did not show a declining trend, with FY2019 showing the highest rate among the surveyed years. Mushroom detection rates increased modestly after FY2015—when sampling focused on mushrooms and wild edible plants—but the FY2019 detection rate was the lowest observed during the eight-year period.

**Fig. 4.**
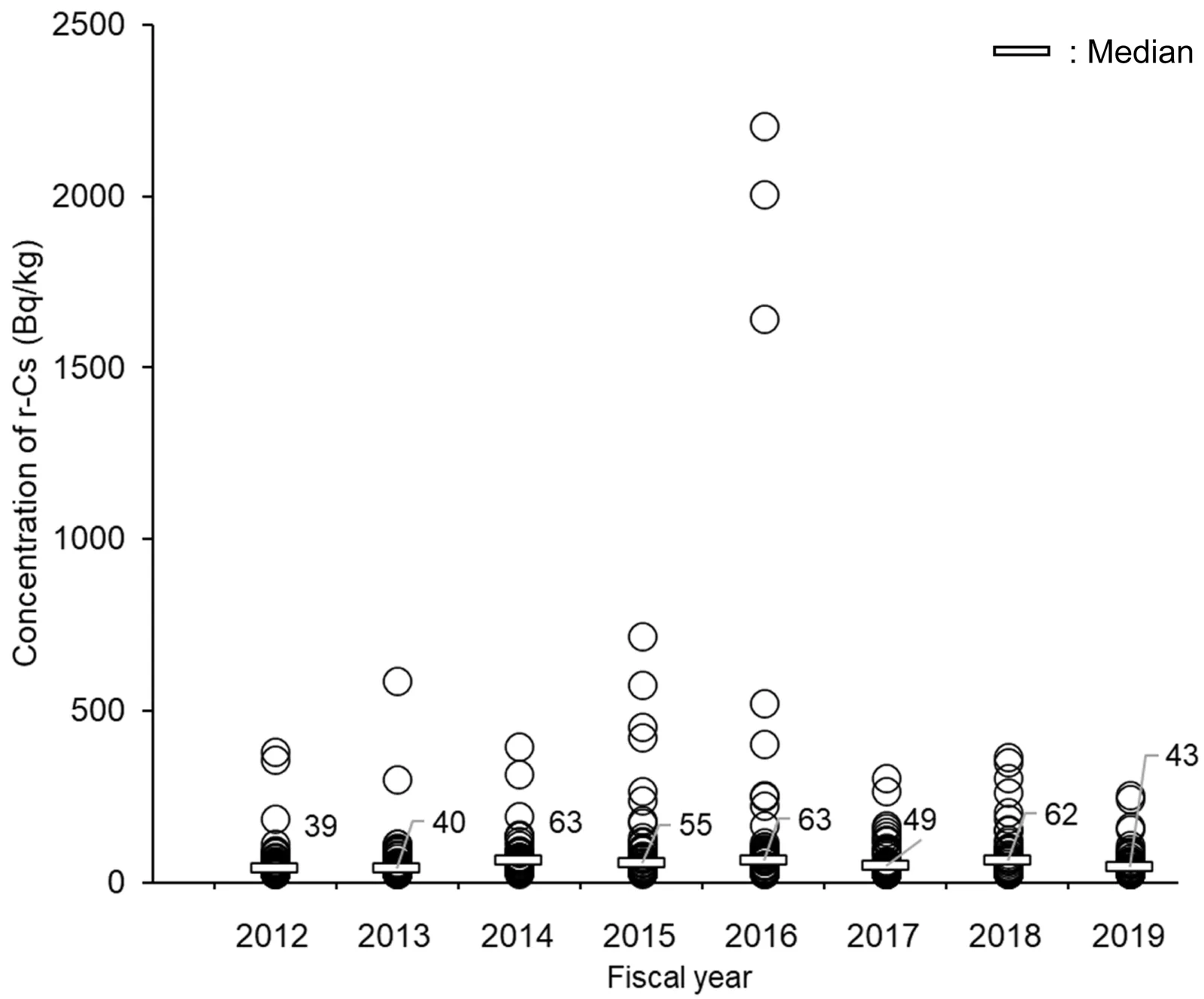
Annual trends in r-Cs concentrations and their median in detected samples

Because the survey methodology relied on purchasing foods distributed in the market, variability in sample availability, climatic conditions, and harvesting seasons caused substantial year-to-year fluctuations in the types and origins of wild-derived foods. Therefore, direct comparison of detected concentrations across years is difficult. Figure 4 summarizes detected r-Cs concentrations and median values. Most positive samples contained less than 1,000 Bq/kg; however, three samples exceeding 1,500 Bq/kg were identified in FY2016. Median concentrations have remained consistently around 50–60 Bq/kg since targeted monitoring began. In FY2024, a natural mushroom sample containing 1,000 Bq/kg r-Cs was reported in official monitoring results, reinforcing the need for continued surveillance of wild foods^16^^)^.

Cultivated vegetables, fruits, and livestock products maintained low detection rates throughout the survey period, indicating sustained effectiveness of contamination-reduction measures implemented after the accident. The elevated detection rate for meat and eggs in FY2015 resulted from detections in several wild bird and wild animal meat samples, which constituted a small sample set. No r-Cs was detected in general livestock products other than wild game meat.

The proportion of samples exceeding the JML increased to 1.3% in FY2015 due to targeted sampling of mushrooms and wild edible plants. This proportion remained relatively stable in FY2016–2018 (1.3–1.5%) before decreasing to 0.7% in FY2019, partly reflecting reduced availability of wild edible plants during the COVID-19 pandemic. In FY2023, the proportion of exceedances increased to 2.0% (Ministry of Health, Labour, and Welfare; unpublished), illustrating the pronounced influence of natural factors such as weather conditions and sampling season on contamination levels in wild foods.

From FY2012 to FY2019, 62 samples exceeded the JML (Table 2). Koshiabura (*Chengiopanax sciadophylloides*) was the most frequently affected (19 samples), followed by log-grown shiitake mushrooms (*Lentinula edodes*) and related dried products (12 samples), sakura-shimeji (*Hygrophorus russula*)(6 samples), and koutake mushrooms (*Sarcodon aspratus*) and dried koutake products (5 samples). Among wild edible plants, exceedances were limited to koshiabura (*Chengiopanax sciadophylloide*), aralia sprouts (*Aralia elata*), and bracken (*Pteridium aquilinum*) (including bracken-derived products). Twelve mushroom types exceeded the JML, nine of which were wild mushrooms. Due to effective management of cultivated log-grown mushrooms, exceedances have markedly decreased and have been limited primarily to dried processed products (e.g., dried mushroom powder) since around 2015. Since around 2020, exceedances have occurred almost exclusively in wild mushrooms and wild edible plants^17^^,^^18^^,^^19^^,^^20^^,^^21^^,^^22^^)^. Because these foods remain culturally significant and widely consumed in Japan, focused monitoring continues to play an essential role in preventing the distribution of highly contaminated products. Furthermore, measurement of r-Cs in wild foods also provides valuable information on contamination conditions in forest ecosystems.

**Table 2.** Breakdown of samples exceeding JMLs in the 2012–2019 Surveys

**Food category**	**Fiscal year exceeded JML**	**Item**	**Number of exceeded JML**
Wild edible plants	2014, 2016-2019	Koshiabura (*Chengiopanax sciadophylloides*)	19
	2013, 2016	Bracken *(Pteridium aquilinum)* and its dried products	3
	2014, 2018	Aralia sprout (*Aralia elata*)	2
Mushrooms	2012-2015, 2017-2019	Log-grown shiitake (*Lentinula edodes*) and its dried products	12
	2014-2018	Sakura-shimeji (*Hygrophorus russula*)	6
	2015,2017-2018	Koutake (*Sarcodon aspratus*) and its dried products	5
	2014-2015	Apricot milkcap (*Lactifluus volemus*)	2
	2012-2013	Nameko (*Pholiota microspora*)	2
	2016	Hatsutake (*Lactarius lividatus*)	2
	2015, 2018	Maitake (*Grifola frondosa*), dried products	2
	2016	Akayamadori (*Leccinum extremiorienta*)	1
	2015	Brick cap (*Hypholoma lateritium*)	1
	2015	Kurokawa (*Boletopsis grisea*)	1
	2015	Purple-staining Cortinarius (*Cortinarius* spp.)	1
	2016	Blewit (*Lepista nuda*)	1
Meat, eggs	2015	Wild boar meat (*Sus scrofa*)	1
Others	2012	Mulberry tea leaves (*Morus alba*)	1
		**Total**	**62**

JML: Japanese maximum level for radioactive cesium in foods.

### 2.3. r-Cs Concentration in Infant Foods

Monitoring of r-Cs in infant foods has been conducted continuously since FY2012 to address concerns regarding the potentially higher radiosensitivity of infants, for whom a stricter JML of 50 Bq/kg was established—half that for general foods. Because pre-shipment testing by local governments rarely included infant foods, market-based purchasing surveys were implemented to clarify the actual status of r-Cs contamination in domestically produced infant foods^11^^)^.

**Fig. 5.**
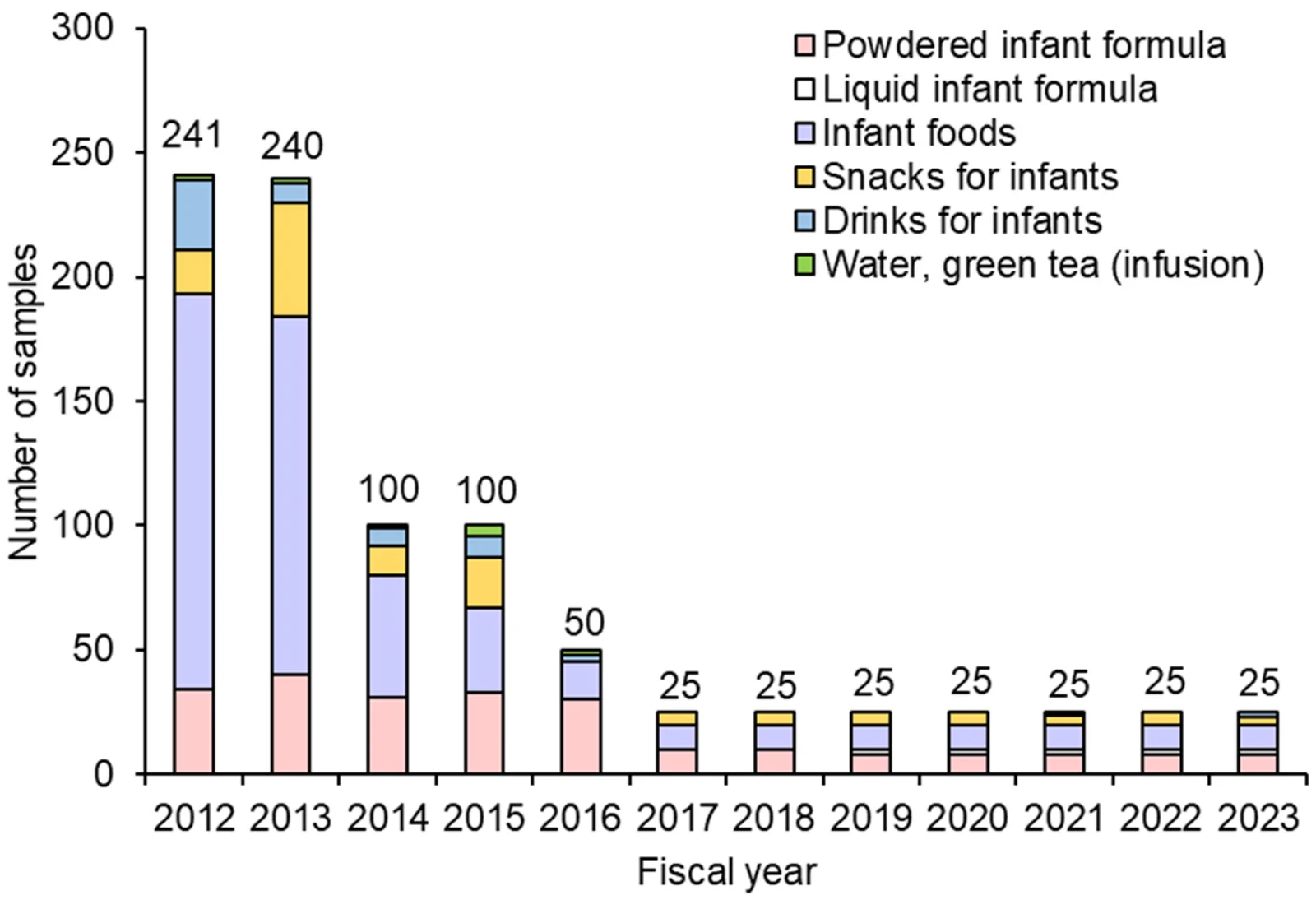
Trends in the number and types of infant food items surveyed

The number of samples surveyed each year was 240 in FY2012–2013, 100 in FY2014–2015, 50 in FY2016, and 25 per year from FY2017 onward (Fig. 5). From FY2012 to FY2023, a total of 906 samples were analyzed, including 228 powdered formula samples, 10 liquid formula samples (survey initiated in FY2019 following domestic commercialization), 471 baby food items (staples and main dishes), 128 snacks for infants, 58 beverages for infants, and 11 drinking water or green tea samples for infants. A stricter JML of 10 Bq/kg applies to infant drinking water and green tea, while all other infant food products must meet the 50 Bq/kg standard.

The detection limits for each product category by fiscal year are summarized in Table 3. All products were analyzed below their target detection limits, and no r-Cs levels above the detection limit were observed in any of the 906 samples tested between FY2012 and FY2023. These results demonstrate that manufacturers have consistently controlled ingredient sourcing and production processes to prevent contamination of infant food products since the Fukushima accident.

**Table 3.** Limit of detection (LOD) for each infant food item

Food categores		Infant foods																								Drinking water			
Target detection limit		5 Bq/kg																								1 Bq/kg			
Fiscal year	Powdered infant formula					Liquid infant formula					Infant foods					Snacks for infants					Drinks for infants					Water, green tea (infusion)			
	No. of sample	Range of LOD				No. of sample	Range of LOD				No. of sample	Range of LOD				No. of sample	Range of LOD				No. of sample	Range of LOD				No. of sample	Range of LOD		
2012	34	2.1	~	3.8		0*	―				159	1.4	~	5		18	1.7	~	3.7		28	0.39	~	0.48		2	0.46	~	0.5
2013	40	3	~	4.9		0*	―				144	2.8	~	4.9		46	3	~	4.9		8	3.6	~	4.5		2	0.72	~	0.97
2014	31	2.8	~	4.7		0*	―				49	2.3	~	4.2		12	2.8	~	3.9		7	3.6	~	4		1	0.16		
2015	33	2	~	3.1		0*	―				34	2.1	~	3.1		20	2	~	3.9		9	2.2	~	3.2		4	0.1	~	0.13
2016	30	2	~	3.1		0*	―				15	2.2	~	2.8		0	-*				3	2.5	~	2.7		2	0.1	~	0.11
2017	10	2.2	~	3.7		0*	―				10	2.1	~	3.9		5	1.5	~	3.4		0	―				0	―		
2018	10	1.7	~	2.2		0*	―				10	1.6	~	2.7		5	1.8	~	2.5		0	―				0	―		
2019	8	1.7	~	2.3		2	2.1	~	2.2		10	1.8	~	2.5		5	1.4	~	2.1		0	―				0	―		
2020	8	1.5	~	2.4		2	0.85	~	0.93		10	0.98	~	1.3		5	1.1	~	2.2		0	―				0	―		
2021	8	1.4	~	1.7		2	0.92	~	1		10	0.82	~	1.2		4	0.83	~	2.1		1	0.98				0	―		
2022	8	1.4	~	2.1		2	0.96	~	1		10	0.93	~	1.1		5	0.92	~	2.5		0	―				0	―		
2023	8	1.5	~	2		2	0.95	~	0.97		10	0.79	~	1.8		3	1.4	~	2.1		2	0.96	~	0.97		0	―		

*: Not applicable, as no relevant products were commercially available at the time of the survey

―: indicates item not surveyed.

Comparable surveys conducted in Europe reported similar detection limits for ^137^Cs in 25 infant foods (0.4–1.7 Bq/kg), and no detectable ^137^Cs was found^23^^)^. This suggests that the contamination status of Japanese infant foods is equivalent to that observed internationally.

Although no formula or liquid milk samples showed detectable r-Cs, the committed effective dose for infants was estimated under the conservative assumption that r-Cs was present at concentrations equal to the maximum detection limits observed in this study (2.7 Bq/kg for ^134^Cs and 2.6 Bq/kg for ^137^Cs) and using the maximum recommended daily intake of formula milk (approximately 130 g/day). Under these assumptions, the committed effective dose from formula milk was estimated at 0.0059 mSv/year—far below the regulatory basis of 1 mSv/year—and indicates that the contribution of infant formula or liquid milk to internal exposure is negligible.

These findings consistently demonstrate that infant foods have remained free of detectable r-Cs contamination for more than a decade after the accident. The strict control of raw materials and production processes by manufacturers, together with continuous monitoring, has ensured that the risk posed to infants via food consumption remains extremely low.

### 2.4. Summary of Commercial Food Monitoring

Previous surveys of r-Cs concentrations in commercial foods consistently indicate that production controls and pre-shipment inspections implemented by local governments and producers have been highly effective for general foods. For cultivated agricultural products, livestock products, and other foods for which cultivation and feeding management are feasible, the likelihood of exceeding the JMLs is extremely low.

Similarly, surveys of infant foods demonstrate that r-Cs concentrations in formula milk and other products for infants—which represent the primary sources of nutrition during early life—are very strictly controlled. Across a wide range of infant food products, the probability of exceeding the JML is considered to be extremely low.

In contrast, naturally derived foods that are difficult to cultivate or manage, such as wild mushrooms and wild edible plants, continue to show occasional exceedances of the JML, and some items still exhibit r-Cs concentrations substantially higher than the limit for general foods. These findings highlight that, while the overall safety of commercial foods is well maintained, careful and continuous monitoring of natural foods remains essential.

It should also be noted that the low frequency of exceedances in commercial foods is partly attributable to regulatory measures, including restrictions on the distribution of foods exceeding the JML. In certain regions, such as restricted zones, some locally produced foods are not placed on the market due to these measures.

## 3. Total Diet Study (TDS) for Estimating Internal Exposure

### 3.1. Overview of the MB-based TDS Method

As noted above, the current regulation values for radioactive materials in foods are designed to ensure that the annual internal dose from food ingestion does not exceed 1 mSv/year for additional exposure from artificial radionuclides (excluding naturally occurring radionuclides such as potassium-40). To verify the appropriateness of this standard, it is essential to estimate the actual intake of radionuclides through the total daily diet and to evaluate how administrative measures implemented after the Fukushima accident have influenced dietary exposure. Because radionuclide concentrations in foods can increase or decrease depending on cooking and processing procedures^24^^,^^25^^,^^26^^,^^27^^,^^28^^)^, the TDS using the MB method has been considered an effective approach, allowing assessment of radionuclide intake after foods have undergone typical preparation.

In September 2011, six months after the accident, a continuous TDS program was initiated using the MB method^29^^,^^30^^,^^31^^,^^32^^)^. In this program, foods consumed by individuals aged one year or older were aggregated using data from the National Health and Nutrition Survey, and approximately 200 food items were collected in each region. These foods were grouped into 14 categories (Table 4), cooked simply as needed, and homogenized to create representative MB samples reflecting actual dietary patterns. Surveys began with three regions in 2011 and later expanded to 12–15 regions, with sampling conducted twice yearly (March and September). Locally produced foods were prioritized to capture regional impacts of the accident. In addition, for MB sample preparation, fresh food items produced within the respective survey regions were obtained whenever possible in order to appropriately reflect regional radiological exposure through the diet.

**Table 4.** List of food groups and representative items included in the MB sample

**No.**	**Food Group**	**Examples**
1	Rice	Polished rice, mochi, etc.
2	Grains & Tubers	Bread, noodles, wheat flour, corn, potatoes, peanuts, etc.
3	Sweets & Sugar	Sugar, manju, cake, candy, chocolate, potato chips, etc.
4	Fats & Oils	Butter, margarine, salad oil, lard, etc.
5	Beans & Processed Beans	Boiled soybeans, tofu, natto, azuki beans, etc.
6	Fruits	Strawberries, apples, kiwifruit, mandarins, etc.
7	Colored Vegetables	Tomatoes, carrots, spinach, pumpkin, etc.
8	Other Vegetables/Mushrooms/Seaweed	Cabbage, cucumber, daikon, shiitake, wakame, etc.
9	Beverages	Sake, beer, wine, green tea, coffee, soft drinks, etc.
10	Seafood	Horse mackerel, flounder, tuna, squid, shrimp, scallops, fish sausage, etc.
11	Meat & Eggs	Beef, pork, chicken, eggs, etc.
12	Dairy Products	Milk, yogurt, cheese, fresh cream, etc.
13	Seasonings	Soy sauce, salt, miso, curry roux, etc.
14	Drinking Water	Tap water

Concentrations of r-Cs (^134^Cs and ^137^Cs) and naturally occurring potassium-40 (^40^K) have been measured in MB samples twice per year using a germanium semiconductor γ-ray spectrometer. Approximately 2 kg of homogenized samples were placed in 2 L Marinelli containers and measured for 22 hours. Efficiency calibration was carried out using a mixed-nuclide standard source, and data analysis followed the “Radioactivity Measurement Series 7” protocol^33^^)^. The limits of detection (LOD) were typically 0.03–0.06 Bq/kg for ^134^Cs and ^137^Cs and 0.5–1.5 Bq/kg for ^40^K, and values were decay-corrected to the date of sample preparation.

For ^90^Sr, measurements have been conducted once annually since September 2013 in six regions. After ashing MB samples (excluding drinking water), ^90^Sr was quantified by ion-exchange and iron hydroxide co-precipitation methods described in “Radioactivity Measurement Series 2”^34^^)^. Depending on the food group, the LOD ranged from 0.004 to 0.04 Bq/kg, and concentrations were decay-corrected.

Daily intake of each radionuclide (Bq/person/day) was calculated by multiplying the measured concentration in each food group by its corresponding daily consumption amount. When concentrations were below the LODs, half of the LOD was used. Annual committed effective dose (mSv/year) was then estimated by multiplying daily intake by 365 and using dose coefficients for adults (^134^Cs: 1.9×10^−5^, ^137^Cs: 1.3×10^−5^, ^40^K: 6.2×10^−6^, ^90^Sr: 2.8×10^−5^ mSv/Bq)^35^^)^.

In this section, we review the estimated daily intake of r-Cs, ^90^Sr, and ^40^K and the committed effective doses derived from TDS-MB surveys conducted over more than a decade following the Fukushima accident.

### 3.2. Detection Rate of Radionuclides in MB Samples

r-Cs and ^40^K were measured in 42 MB samples (14 food groups ×3 regions) in September 2011, 168 MB samples (14 food groups ×12 regions) in March 2012, and 210 MB samples (14 food groups ×15 regions) after September 2012. ^90^Sr was measured in 84 MB samples (14 food groups ×6 regions) in September 2013. The overall detection rates of ^134^Cs, ^137^Cs, ^40^K and ^90^Sr in each survey (number of detected samples/number of measured samples ×100 (%)) are shown inFigure** 6**. The detection rates in September 2011, six months after the accident, were about 60% for both ^134^Cs and ^137^Cs. After that, the detection rate of ^134^Cs was remarkably decreased by September 2015, and it was not detected in any food group in any region after September 2018. Alternately, the detection rate of ^137^Cs decreased more slowly than that of ^134^Cs, and after March 2015 the detection rate was 30–40%. The half-life of ^134^Cs is about two years, and that of ^137^Cs is about 30 years^36^^)^. When the abundance at the time of the accident in March 2011 was set to ‘1’, the theoretical residual ratios based on the half-lives of ^134^Cs and ^137^Cs are approximately 0.5 and 0.06 for ^134^Cs in March 2013 and March 2019, respectively, and approximately 0.95 and 0.83 for ^137^Cs in the same years. The difference between the residual ratios increased over time. In this survey, it was considered that the number of samples in which ^134^Cs was detected decreased over time due to its short half-life, resulting in an increasing difference in detection rates between ^134^Cs and ^137^Cs as time passed since the accident. For ^90^Sr, the detection ratio remained relatively constant at around 50% from the beginning of the survey in September 2013 to the survey in September 2018. The detection ratio of ^40^K remained constant at about 90% in all survey periods. In most MB samples, ^40^K was not detected in drinking water of Group 14. In addition, although ^40^K was not detected in some samples of Group 1 and Group 4, it was detected in all samples of the other food groups, confirming that ^40^K is contained in most foods that Japanese people consume on a daily basis.

**Fig. 6.**
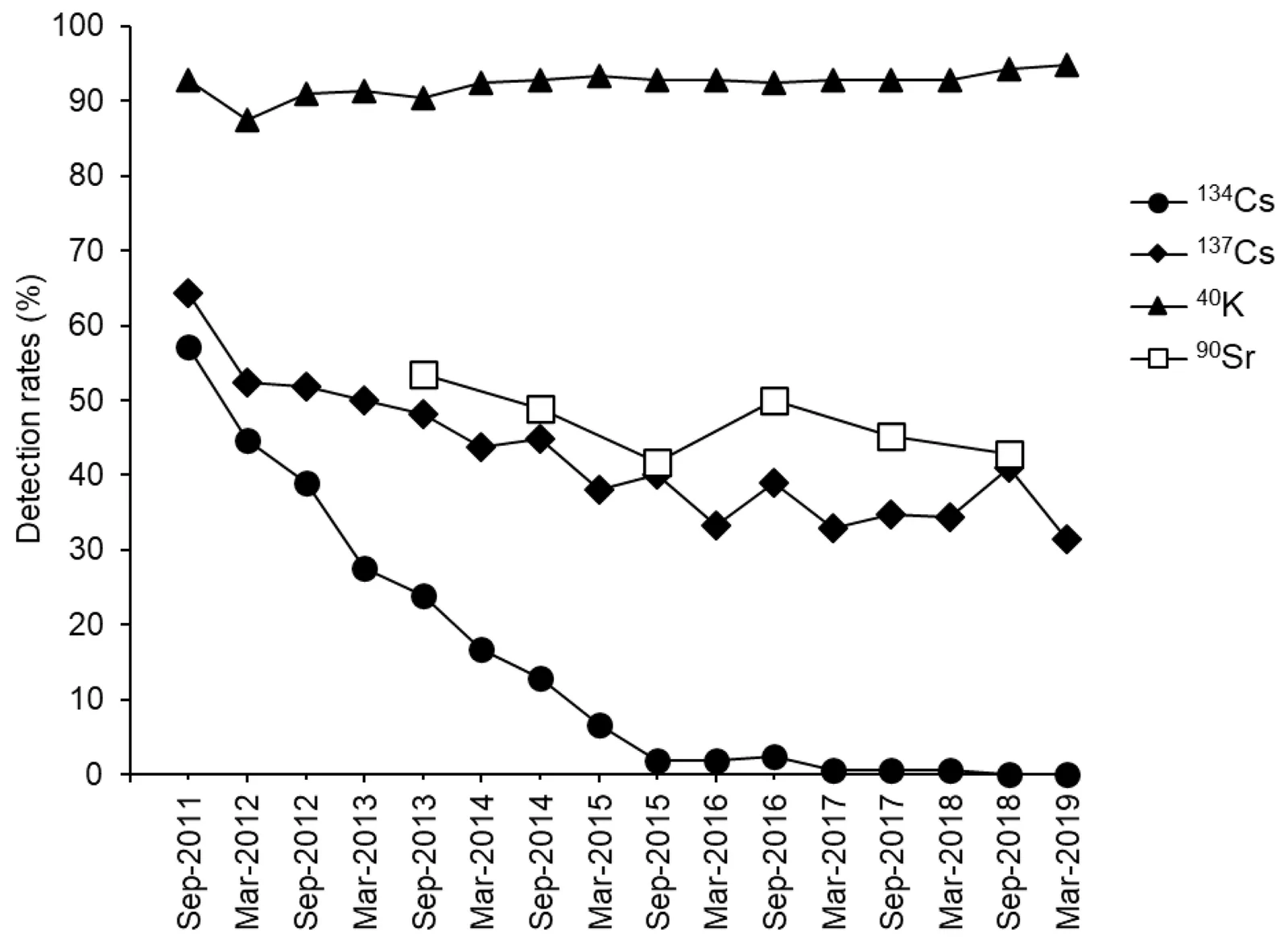
Time-dependent changes in the detection rates of radionuclides in MB samples

**Fig. 7.**
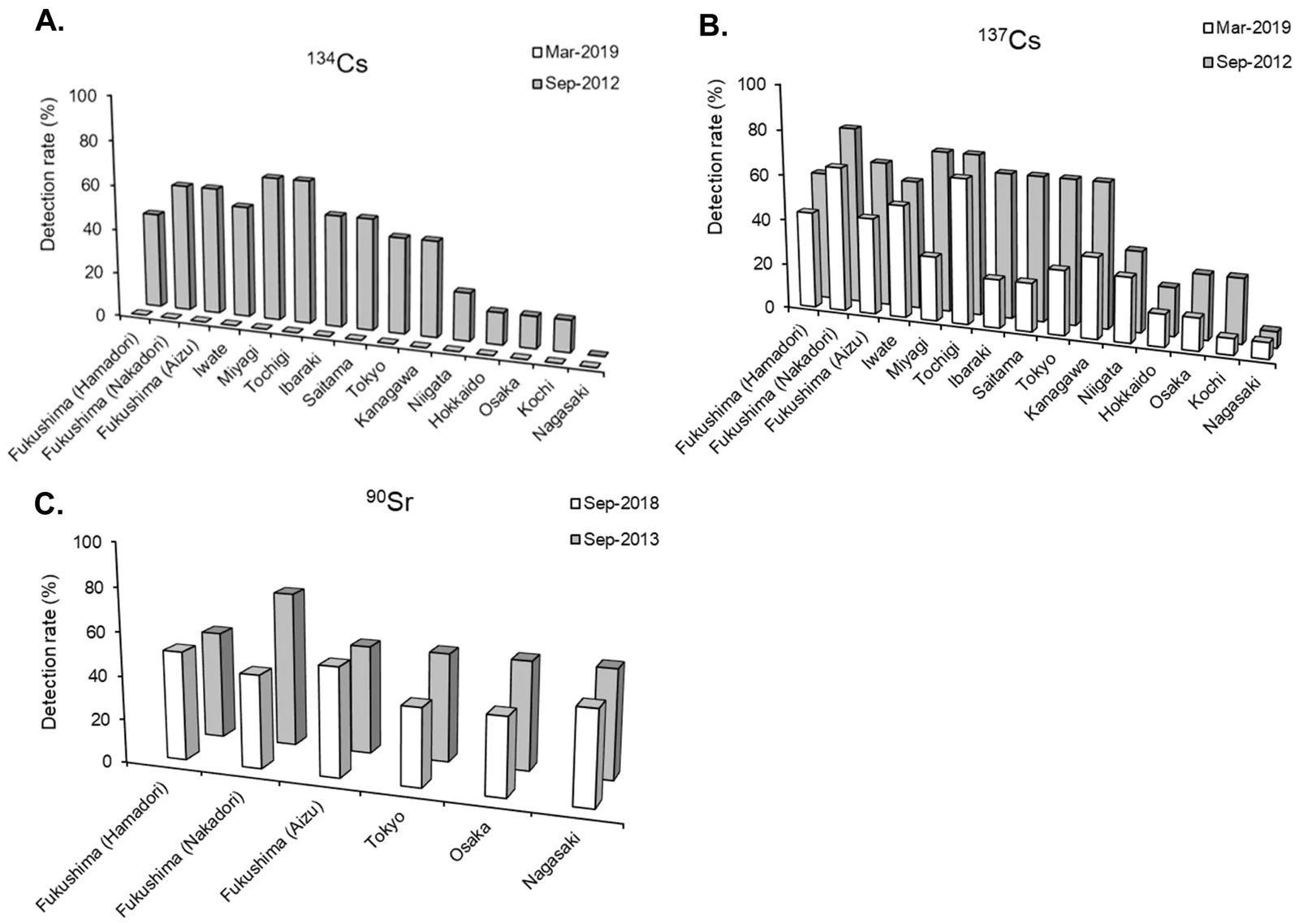
Regional detection rates of ^134^Cs (A), ^137^Cs (B), and ^90^Sr (C) in MB samples

Figure 7A and 7B show the regional detection ratios of ^134^Cs and ^137^Cs in the September 2012 surveys, which were conducted in 15 regions, and in September 2019, the latest published data^31^^)^. Figure 7C shows the regional detection ratios of ^90^Sr in the first surveys in September 2013, and in September 2018, the latest published data^32^^)^. The detection ratio of ^134^Cs in the September 2012 surveys (Fig. 7A) was about 40–65% in Fukushima Prefecture and neighboring Tohoku region (e.g. Miyagi and Iwate) and Kanto region (e.g., Tochigi, Ibaraki, and Saitama), while it ranged from about 0–15% in distant regions (Hokkaido, Osaka, Kochi, and Nagasaki), which are considered to have been less affected by the Fukushima accident. Regional differences in the ^134^Cs detection ratio gradually decreased with the passage of time, and eventually reached 0% in all survey regions in September 2019. Similar to ^134^Cs, the detection rate of ^137^Cs (Fig. 7B) showed large regional differences in the September 2012 surveys, with approximately 60–70% in Fukushima Prefecture and neighboring regions, and approximately 10–35% in distant regions. The detection rate of ^137^Cs in the March 2019 surveys was approximately 20–65% in Fukushima Prefecture and neighboring regions, and 10–30% in distant regions. Although regional differences were still observed in the detection rate of ^137^Cs, it was confirmed that the detection rate of ^137^Cs had decreased remarkably in the Kanto region (e.g., Ibaraki, Saitama, and Tokyo), and the difference between the Kanto region and distant regions had become small.

No clear regional differences were observed in the detection rate of ^90^Sr in the September 2013 surveys.

The detection rate of ^90^Sr was 71% in Nakadori (Fukushima Prefecture) and approximately 50% in other regions of Fukushima Prefecture, Tokyo, and more distant regions such as Osaka and Nagasaki in the September 2013 surveys (Fig. 7C). In the September 2018 surveys, the detection rate ranged from 35% to 50%, showing a slight overall decrease.

Despite these variations, no clear regional differences in ^90^Sr detection rates related to distance from the Fukushima Daiichi Nuclear Power Plant (FDNPP) have been observed, in contrast to the pattern observed for r-Cs. These results suggest that the levels of ^90^Sr in food did not exceed the background levels resulting from fallout of past atmospheric nuclear tests, making it difficult to conclude that ^90^Sr detected in our surveys originated from the Fukushima accident.

### 3.3. Estimated Daily Intake and Annual Committed Effective Dose

The daily intake (Bq/person/day) and annual dose (mSv/year) of r-Cs estimated in the surveys from September 2011 to March 2019 are shown in **Table 5A**. The daily intake of r-Cs was 0.085–3.4 Bq/person/day and the annual dose was 0.00049–0.019 mSv/year. The highest annual dose was observed in Fukushima Prefecture (Nakadori) in September 2011. Considering the impact of the Fukushima accident, it was considered reasonable that the annual dose in Fukushima Prefecture reached the highest level six months after the accident. However, the dose was only 2% of 1 mSv/year, the upper limit of the dose received from food, which was considered sufficiently low.

**Table 5A.** Trends in daily intake and annual committed effective dose of r-Cs (A), ^90^Sr (B), and ^40^K (C) estimated by the MB method

	r-Cs (^134^Cs + ^137^Cs)															
	Sep-2011	Mar-2012	Sep-2012	Mar-2013	Sep-2013	Mar-2014	Sep-2014	Mar-2015	Sep-2015	Mar-2016	Sep-2016	Mar-2017	Sep-2017	Mar-2018	Sep-2018	Mar-2019
Fukushima (Hamadori)		1.1	0.33	1.3	0.46	0.35	0.42	0.29	0.25	0.17	0.20	0.18	0.16	0.17	0.16	0.13
		0.0063	0.0018	0.0071	0.0027	0.0019	0.0022	0.0016	0.0013	0.00092	0.0011	0.00098	0.00088	0.00090	0.00086	0.00071
Fukushima (Nakadori)	3.4	1.2	0.68	0.97	0.49	0.35	0.30	0.37	0.29	0.18	0.23	0.18	0.16	0.21	0.21	0.19
	0.019	0.0066	0.0037	0.0054	0.0027	0.0019	0.0016	0.0020	0.0015	0.00096	0.0012	0.00095	0.00083	0.0011	0.0011	0.00099
Fukushima (Aizu)		0.69	0.42	0.79	0.33	0.31	0.20	0.19	0.17	0.18	0.17	0.16	0.16	0.13	0.19	0.15
		0.0039	0.0038	0.0048	0.0018	0.0017	0.0011	0.0010	0.00090	0.00098	0.00093	0.00090	0.00086	0.00071	0.00097	0.00080
Iwate		1.7	0.51	0.47	0.38	0.32	0.21	0.19	0.17	0.19	0.18	0.15	0.22	0.14	0.12	0.14
		0.0094	0.0040	0.0035	0.0021	0.0017	0.0011	0.0010	0.00093	0.0010	0.0010	0.00083	0.0011	0.00076	0.00067	0.00079
Miyagi	0.20		0.18	0.21	0.20	0.19	0.19	0.17	0.18	0.19	0.18	0.19	0.18	0.18	0.18	0.19
	0.018		0.0057	0.0033	0.0025	0.0012	0.0013	0.00098	0.00091	0.00076	0.0010	0.00092	0.00085	0.0011	0.00076	0.00064
Tochigi		0.18	0.19	0.19	0.18	0.17	0.17	0.17	0.17	0.17	0.18	0.18	0.18	0.18	0.18	0.17
		0.0090	0.0032	0.0030	0.0015	0.0013	0.0012	0.00093	0.00089	0.0011	0.0014	0.0010	0.00075	0.00075	0.00098	0.00083
Ibaraki		0.19	0.20	0.20	0.18	0.18	0.18	0.16	0.16	0.18	0.17	0.18	0.18	0.17	0.17	0.18
		0.0044	0.0035	0.0038	0.0014	0.0012	0.0012	0.00087	0.0011	0.00078	0.0011	0.00082	0.00073	0.00086	0.00071	0.00054
Saitama		0.18	0.17	0.18	0.17	0.17	0.17	0.16	0.16	0.16	0.17	0.18	0.18	0.17	0.17	0.17
		0.0039	0.0024	0.0016	0.0013	0.00086	0.0014	0.00086	0.0011	0.00072	0.00086	0.00090	0.00069	0.00065	0.00063	0.00070
Tokyo	0.18		0.19	0.19	0.17	0.17	0.17	0.17	0.16	0.16	0.17	0.18	0.17	0.17	0.17	0.19
	0.0024		0.0022	0.0014	0.00085	0.00099	0.0010	0.00075	0.00064	0.00081	0.00080	0.00069	0.00084	0.00064	0.00074	0.00058
Kanagawa		0.16	0.17	0.18	0.17	0.17	0.16	0.16	0.15	0.17	0.18	0.18	0.17	0.18	0.18	0.17
		0.0033	0.0021	0.0013	0.0010	0.0011	0.0013	0.0011	0.00070	0.00077	0.0010	0.00076	0.00065	0.00064	0.00062	0.00073
Niigata		0.17	0.19	0.18	0.18	0.18	0.19	0.18	0.17	0.18	0.18	0.18	0.18	0.18	0.17	0.18
		0.0023	0.0017	0.0018	0.0010	0.00084	0.0010	0.00073	0.00070	0.00065	0.00082	0.00067	0.00059	0.00064	0.00061	0.00059
Hokkaido		0.16	0.15	0.16	0.16	0.16	0.16	0.16	0.16	0.16	0.17	0.16	0.16	0.17	0.16	0.16
		0.00095	0.00095	0.0010	0.00083	0.00091	0.00070	0.00066	0.00123	0.00072	0.00074	0.00070	0.00063	0.00060	0.00064	0.00053
Osaka		0.16	0.15	0.15	0.16	0.17	0.16	0.16	0.15	0.16	0.16	0.17	0.17	0.15	0.16	0.16
		0.0016	0.0012	0.00085	0.00099	0.00076	0.00069	0.00062	0.00071	0.00072	0.00068	0.00066	0.00057	0.00052	0.00060	0.00054
Kochi		0.18	0.14	0.15	0.15	0.15	0.15	0.15	0.14	0.15	0.17	0.17	0.18	0.17	0.16	0.16
		0.0012	0.0013	0.00093	0.00084	0.00094	0.00072	0.00061	0.00072	0.00062	0.00071	0.00075	0.00056	0.00057	0.00057	0.00051
Nagasaki			0.14	0.15	0.14	0.15	0.15	0.15	0.15	0.15	0.16	0.17	0.17	0.17	0.16	0.16
			0.00088	0.0010	0.00084	0.00074	0.00068	0.00065	0.00061	0.00066	0.00066	0.00064	0.00058	0.00050	0.00056	0.00049

For each region, the upper value represents the daily intake of r-Cs in Bq/day, while the lower value corresponds to the annual committed effective dose in mSv/year

In cases where concentrations in MB samples were reported as ND, a value equal to half the limit of detection (1/2LOD) was used to estimate the annual intake and the annual committed effective dose.

**Table 5B.** 

	^90^Sr					
	Sep-2013	Sep-2014	Sep-2015	Sep-2016	Sep-2017	Sep-2018
Fukushima (Hamadori)	0.074	0.043	0.046	0.058	0.042	0.035
	0.00076	0.00044	0.00047	0.00059	0.00043	0.00036
Fukushima (Nakadori)	0.052	0.042	0.057	0.042	0.045	0.040
	0.00053	0.00043	0.00058	0.00043	0.00046	0.00041
Fukushima (Aizu)	0.047	0.041	0.041	0.039	0.045	0.044
	0.00048	0.00042	0.00042	0.00040	0.00046	0.00044
Tokyo	0.047	0.034	0.035	0.035	0.031	0.040
	0.00049	0.00034	0.00036	0.00036	0.00032	0.00041
Osaka	0.043	0.059	0.044	0.041	0.041	0.040
	0.00044	0.00060	0.00045	0.00042	0.00042	0.00041
Nagasaki	0.054	0.040	0.041	0.042	0.035	0.038
	0.00055	0.00040	0.00042	0.00043	0.00035	0.00039

For each region, the upper value represents the daily intake of r-Cs in Bq/day, while the lower value corresponds to the annual committed effective dose in mSv/year

In cases where concentrations in MB samples were reported as ND, a value equal to half the limit of detection (1/2LOD) was used to estimate the annual intake and the annual committed effective dose.

**Table 5C.** 

	^40^K															
	Sep-2011	Mar-2012	Sep-2012	Mar-2013	Sep-2013	Mar-2014	Sep-2014	Mar-2015	Sep-2015	Mar-2016	Sep-2016	Mar-2017	Sep-2017	Mar-2018	Sep-2018	Mar-2019
Fukushima (Hamadori)		82	88	88	89	80	78	79	82	83	80	80	80	78	74	77
		0.19	0.20	0.20	0.20	0.18	0.18	0.18	0.19	0.19	0.18	0.18	0.18	0.18	0.17	0.17
Fukushima (Nakadori)	83	83	87	86	91	85	80	84	79	80	77	78	75	78	77	77
	0.19	0.19	0.20	0.19	0.21	0.19	0.18	0.19	0.18	0.18	0.17	0.18	0.17	0.18	0.17	0.17
Fukushima (Aizu)		79	83	87	84	85	85	78	77	84	80	81	78	76	76	76
		0.18	0.19	0.20	0.19	0.19	0.19	0.18	0.17	0.19	0.18	0.18	0.18	0.17	0.17	0.17
Iwate		89	90	86	91	83	84	79	78	82	78	79	79	81	74	80
		0.20	0.20	0.19	0.21	0.19	0.19	0.18	0.18	0.19	0.18	0.18	0.18	0.18	0.17	0.18
Miyagi	91		81	94	90	83	82	76	81	85	81	85	78	79	80	84
	0.20		0.18	0.21	0.20	0.19	0.19	0.17	0.18	0.19	0.18	0.19	0.18	0.18	0.18	0.19
Tochigi		79	84	82	78	76	76	75	73	75	80	79	81	78	80	76
		0.18	0.19	0.19	0.18	0.17	0.17	0.17	0.17	0.17	0.18	0.18	0.18	0.18	0.18	0.17
Ibaraki		86	84	86	79	80	72	71	72	78	77	78	78	77	77	80
		0.19	0.20	0.20	0.18	0.18	0.18	0.16	0.16	0.18	0.17	0.18	0.18	0.17	0.17	0.18
Saitama		77	77	79	77	74	75	69	69	70	77	80	79	76	75	76
		0.18	0.17	0.18	0.17	0.17	0.17	0.16	0.16	0.16	0.17	0.18	0.18	0.17	0.17	0.17
Tokyo	77		83	84	75	73	74	73	70	72	76	78	77	77	77	82
	0.18		0.19	0.19	0.17	0.17	0.17	0.17	0.16	0.16	0.17	0.18	0.17	0.17	0.17	0.19
Kanagawa		69	77	78	73	74	73	71	68	73	80	79	76	78	79	73
		0.16	0.17	0.18	0.17	0.17	0.16	0.16	0.15	0.17	0.18	0.18	0.17	0.18	0.18	0.17
Niigata		74	83	81	82	81	82	77	77	78	81	81	81	80	76	81
		0.17	0.19	0.18	0.18	0.18	0.19	0.18	0.17	0.18	0.18	0.18	0.18	0.18	0.17	0.18
Hokkaido		70	67	73	71	71	70	72	72	70	73	72	72	75	72	71
		0.16	0.15	0.16	0.16	0.16	0.16	0.16	0.16	0.16	0.17	0.16	0.16	0.17	0.16	0.16
Osaka		71	68	68	70	76	72	70	68	71	72	74	77	67	69	71
		0.16	0.15	0.15	0.16	0.17	0.16	0.16	0.15	0.16	0.16	0.17	0.17	0.15	0.16	0.16
Kochi		78	61	65	66	66	67	64	63	65	76	75	79	74	70	72
		0.18	0.14	0.15	0.15	0.15	0.15	0.15	0.14	0.15	0.17	0.17	0.18	0.17	0.16	0.16
Nagasaki			63	65	62	68	67	68	66	67	70	76	74	76	70	71
			0.14	0.15	0.14	0.15	0.15	0.15	0.15	0.15	0.16	0.17	0.17	0.17	0.16	0.16

For each region, the upper value represents the daily intake of radionuclides in Bq/day, while the lower value corresponds to the annual committed effective dose in mSv/year.

In cases where concentrations in MB samples were reported as ND, a value equal to half the limit of detection (1/2LOD) was used to estimate the annual intake and the annual committed effective dose.

**Fig. 8.**
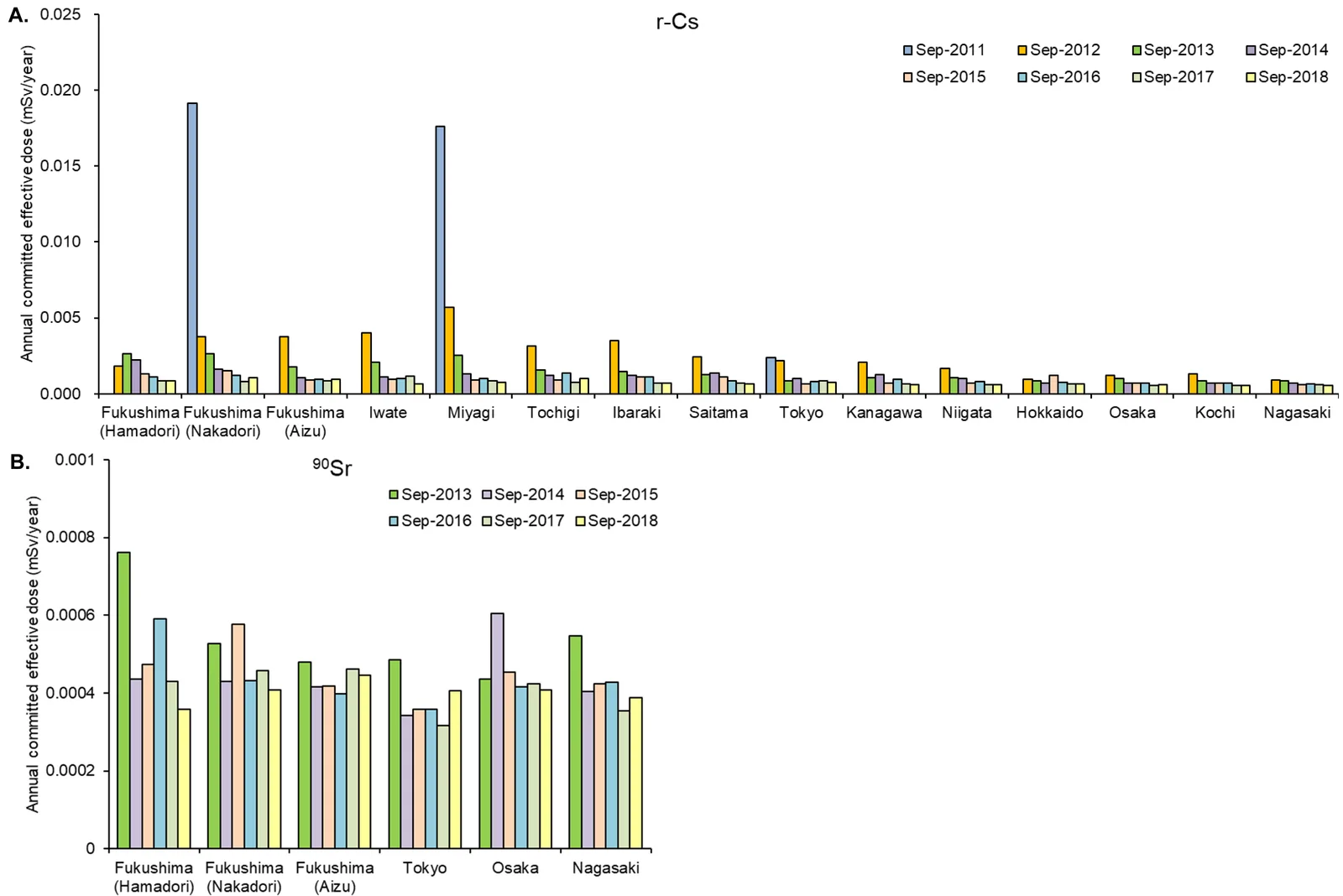
Trends in annual committed effective dose of r-Cs (A) and ^90^Sr (B) estimated by the MB method

Figure 8A shows the changes in the annual dose of r-Cs by region estimated in each September survey since September 2011. Focusing on Fukushima (Nakadori), where continuous surveillance has been conducted, the annual dose decreased sharply since the first survey, and the dose in September 2014 was about a tenth of that in September 2011. In this way, the annual dose in Fukushima Prefecture and neighboring regions decreased sharply three years after the accident, and the difference between Fukushima prefecture and distant regions gradually narrowed over time. In addition, the annual dose decreased nationwide with the passage of time since the accident, and the highest annual dose in September 2018 decreased to 0.0011 mSv/year. This dose is equivalent to about 0.1% of the upper limit, which is a sufficiently low level. As mentioned above, in this investigation, the daily intake and annual dose were estimated by assuming that the concentration of radioactive materials in MB samples, with analytical results below the LOD (ND) was half of the LOD. Therefore, it should be noted that recent investigations may have overestimated r-Cs, especially ^134^Cs, for which the detection rate was low or there was no detection. Factors contributing to the large decreases in r-Cs received from food in the years after the accident include: (i) the natural decay of ^134^Cs and ^137^Cs, (ii) effective and proactive implementation of decontamination measures immediately after the accident, and (iii) administrative measures such as pre-shipment inspections that actively contributed to reducing contamination^37^^,^^38^^)^.

In September 2011 and September 2012, there was a large difference in annual dose of r-Cs from foods between Fukushima Prefecture and its surrounding regions compared to distant regions. In September 2012, the difference between the maximum and minimum values was about 6.5 times. However, the regional difference in the annual dose gradually decreased over time, and in March 2019, the regional difference was reduced to about 2 times. However, in recent years, the downward trend of the annual dose from r-Cs has slowed down, and since March 2017, it has been in a constant range of 0.0005–0.0010 mSv/year. At present, as the effects of the administrative and decontamination measures have stabilized, the main contributor to the decrease in the annual dose of r-Cs in food is the decay of ^137^Cs, as the contribution of ^134^Cs has become negligible due to its shorter half-life. Along with the gradual decay of ^137^Cs, which has a physical half-life of about 30 years, the annual dose from r-Cs is expected to gradually decrease in the future. According to the latest data published on the Consumer Affairs Agency website^39^^)^, the annual dose of r-Cs in the September 2024 surveys was 0.0005–0.0009 mSv/year and has remained almost unchanged since the March 2019 surveys. These values remain slightly higher than reported pre-accident annual doses of ^137^Cs in Japan (up to 0.000378 mSv/year)^40^^)^. It should be noted that direct comparison with pre-accident data is limited due to differences in radionuclide composition (r-Cs vs ^137^Cs), as well as difference in sample preparation, analytical methods, and dose estimation approaches between studies.

**Fig. 9.**
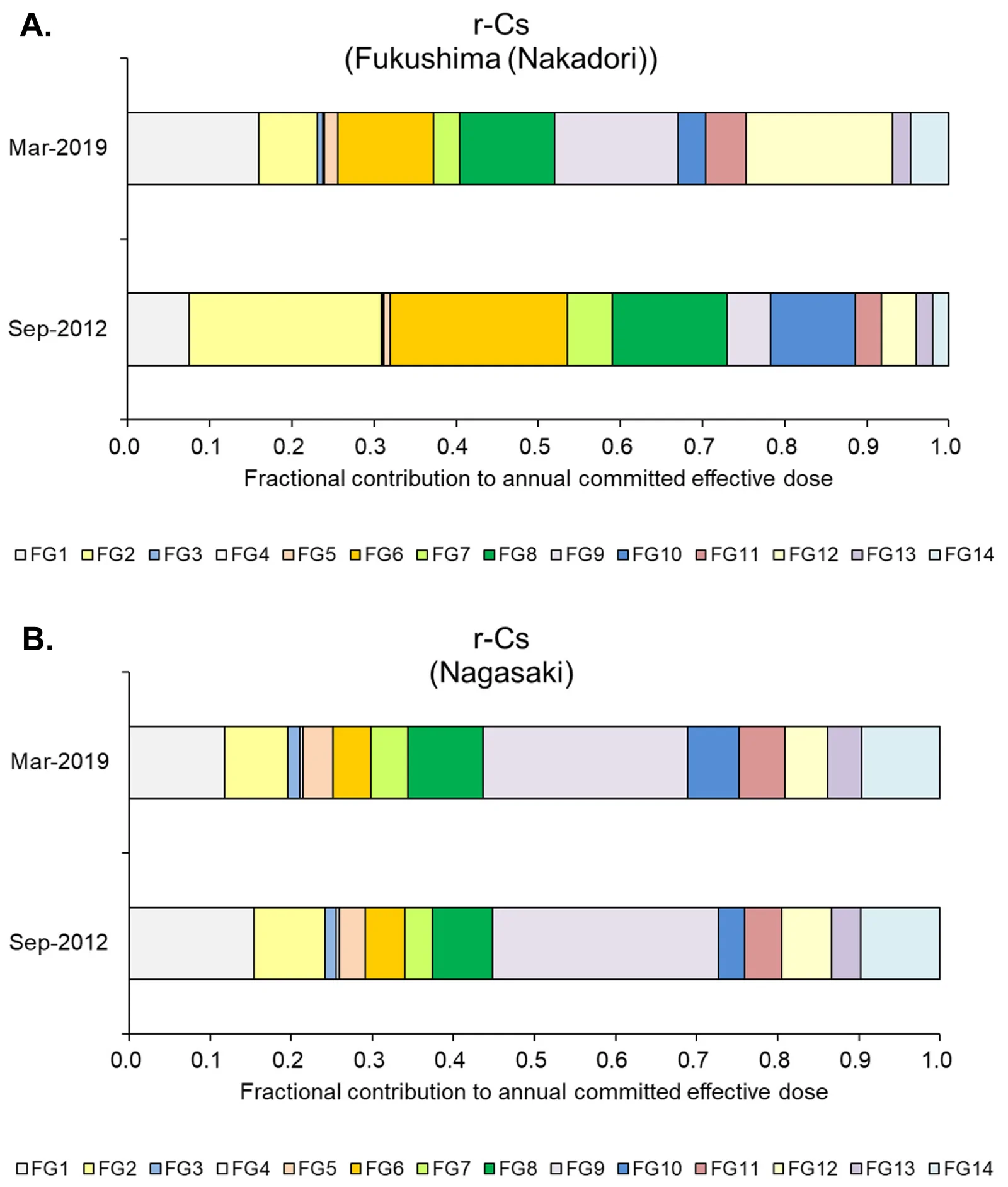
Contribution of each food group to the committed effective dose in Fukushima (Nakadori) (A) and Nagasaki (B)

One of the features of the MB method in a TDS is that it is possible to evaluate the contribution of each food group to the intake. As mentioned above, the annual dose was estimated by assuming that the radioactive material concentration in the MB samples, with analytical results below the LOD (ND) was half of the LOD. As an example, Figure 9A and 9B show the contribution of r-Cs to the annual dose of each food group in Fukushima (Nakadori) and Nagasaki in the September 2012 and March 2019 surveys. In Fukushima Prefecture and neighboring regions, the contribution of Groups 2, 6, 8, and 10 was relatively large for several years after the accident (Fig. 9A). This was thought to reflect that Groups 6, 8, and 10 are food categories in which r-Cs is frequently detected and that these foods are consumed in relatively large quantities. Since around 2019, however, the contributions of Groups 1, 9, and 12 have become relatively large. This is thought to be due to the fact that the ratio of each food group to the total food intake has become more influential than the r-Cs concentration in the food, because the number of food groups in which r-Cs was not detected has increased, and even if r-Cs was detected, the value was close to the LOD. In Nagasaki, where the detection rate and concentration of r-Cs were low from the beginning of the surveys, the contribution ratio of each food group to the annual dose was almost the same between the surveys in September 2012 and September 2019 **(**Fig. 9B), and showed a high similarity with the ratio of each food group to the total food intake from the beginning of the surveys.

The daily intake (Bq/person/day) and annual dose (mSv/year) of ^90^Sr estimated in the surveys from September 2013 to September 2018 are summarized in Table 5B. The daily intake of ^90^Sr estimated throughout the survey period was 0.031–0.074 Bq/person/day, and the annual dose was 0.00032–0.00076 mSv/year. The highest annual dose was observed in Fukushima Prefecture (Hamadori) in the surveys of September 2013; however, the dose was sufficiently low, being less than 0.1% of the upper limit of 1 mSv/year of the dose for food intake.

The annual dose of ^90^Sr estimated by region in the surveys from September 2013 to September 2018 is shown in Figure 8B. In the surveys of September 2013, the annual dose in Fukushima (Hamadori) was estimated to be higher than that in other regions. This was thought to be due to the fact that ^90^Sr above the LOD was detected in Group 1 and Group 9, two groups from which large amounts were consumed. However, there was no clear regional difference in the annual dose of ^90^Sr between Fukushima Prefecture and other regions in the same period. On the contrary, there were cases where the annual dose was estimated to be higher in regions far from the FDNPP. There was no consistent trend between the distance from the FDNPP and the annual dose of ^90^Sr. In addition, the daily intake of ^90^Sr after the Fukushima accident was similar to the results of surveys conducted before the accident^40^^,^^41^^)^. These results, as well as the detection rate, suggest that it is difficult to judge whether the ^90^Sr detected in this survey originated from the Fukushima accident, and that the effects of the Fukushima accident on food products did not exceed the background level of ^90^Sr existing in the environment as a fallout from past atmospheric nuclear tests. According to the latest data published from the Consumer Affairs Agency website^42^^)^, the range of annual doses of ^90^Sr in the surveys conducted in September 2023 was 0.00026–0.00033 mSv/year, which was slightly lower than that in March 2018. Since the physical half-life of ^90^Sr is approximately 30 years^36^^)^, the origin of the detected ^90^Sr was unknown, but it was assumed that the annual dose of ^90^Sr would gradually decrease in the future along with the decay of the physical half-life of ^90^Sr.

The annual dose of ^90^Sr was lower than that of r-Cs in all six regions surveyed during the entire survey period, and the ratio of the annual dose of ^90^Sr to r-Cs in the corresponding survey period and area was in the range of 0.2–0.9. The ratio of the annual dose of ^90^Sr to r-Cs in the surveys conducted in September 2013 was approximately 0.2–0.3 in Fukushima Prefecture and approximately 0.4–0.6 in the other three regions, while the ratio of the annual dose of both nuclides in the surveys conducted in September 2018 was approximately 0.4–0.5 in Fukushima Prefecture and approximately 0.6–0.7 in the other regions. In particular, in Nagasaki, the ratio of the annual dose of ^90^Sr to r-Cs was 0.6–0.7 throughout the entire survey period, and it was confirmed that there was little change. In the three regions in Fukushima Prefecture, the dose of r-Cs decreased annually, while the annual dose of ^90^Sr did not change significantly. Therefore, it is considered that the ratio of the annual dose of ^90^Sr increased with the passage of time after the accident. Therefore, as the annual dose of r-Cs decreases in Fukushima Prefecture, the ratio of the annual dose of ^90^Sr to that of r-Cs is expected to increase further, and in the other three regions, it is expected to remain at 0.6–0.7.

The estimated daily intake (61–94 Bq/person/day) and annual dose (0.14–0.21 mSv/year) of ^40^K from September 2011 to March 2019 are shown in Table 5C. Unlike r-Cs, the daily intake and annual dose of ^40^K did not change with time and remained almost constant. Compared with the annual dose of r-Cs, the annual dose of ^40^K was up to 10 times larger immediately after the accident and up to 300 times larger in recent years. In other words, it was confirmed that the annual dose of r-Cs and ^90^Sr was sufficiently lower than the annual dose of ^40^K, which is a natural radionuclide, in all regions and all periods investigated.

### 3.4. Summary of TDS

The annual doses of r-Cs and ^90^Sr estimated through the MB-based TDS following the Fukushima Daiichi accident have consistently remained far below the regulatory limit of 1 mSv per year, including data obtained in the months immediately after the accident. These long-term datasets demonstrate a marked and sustained decline in dietary exposure to r-Cs, reflecting the combined effects of radioactive decay, food regulations, pre-shipment inspections, and other administrative and environmental measures. Regional differences, initially pronounced between Fukushima and more distant regions, have also narrowed substantially over the years.

For ^90^Sr, both detection rates and annual doses remained extremely low throughout the survey period, suggesting that its presence in food did not exceed background levels associated with historical global fallout. Nevertheless, slightly higher detection rates and doses of r-Cs persist in Fukushima and nearby prefectures—albeit at very low concentrations—highlighting the continued need for targeted monitoring of these regions.

Looking ahead, ongoing monitoring will remain essential in the context of activities such as the release of treated water from the FDNPP beginning in August 2023 and the long-term decommissioning of the facility. Continued TDS implementation will play a crucial role in evaluating potential impacts on the food supply, addressing public concerns, and maintaining domestic and international confidence in Japan’s food safety.

Overall, these long-term monitoring results confirm that Japan’s food safety control system has effectively kept public exposure from food well below international limits established by the Codex Alimentarius Commission and the International Commission on Radiological Protection (ICRP). The integrated approach of regulatory management, environmental remediation, and sustained TDS provides an important model for long-term food safety oversight following a nuclear accident.

## 4. Conclusions

Since the Fukushima accident, Japan has implemented extensive scientific efforts to assess and manage internal exposure from radionuclides in food. This review summarized the findings of long-term monitoring of r-Cs concentrations in commercial foods and the estimation of annual committed effective doses of r-Cs and ^90^Sr using the MB-based TDS.

Surveillance of commercial foods demonstrated that pre-shipment inspections and production controls have been highly effective for cultivated agricultural products and livestock products, resulting in an extremely low probability of detecting r-Cs concentrations exceeding the JML. In contrast, exceedances continue to be observed in wild edible plants, wild mushrooms, and game meat, reflecting the persistence of residual r-Cs in natural environments. These findings underscore the need for sustained, targeted monitoring of wild foods. No r-Cs has been detected in infant foods, indicating that exposure risks for this sensitive population remain negligible. It should also be recognized that the safety of foods on the market is ensured in part by regulatory measures that restrict the distribution of foods exceeding the JML.

The MB-based TDS confirmed that the annual committed effective dose of r-Cs was already below 2% of the 1 mSv/year limit immediately after the accident, despite substantial regional differences at that time. These differences have since diminished markedly, and in recent years annual doses have fallen below 0.001 mSv/year in all regions, demonstrating that dietary exposure to r-Cs is now extremely low nationwide. For ^90^Sr, no clear regional pattern was observed, and the annual doses remained comparable to pre-accident levels, indicating that contributions from the Fukushima accident were minimal and did not exceed background environmental levels.

All of the above findings together show that Japan’s food safety management system for radioactive materials—grounded in science-based regulation, extensive monitoring, and transparent communication—has functioned effectively since the Fukushima accident. Continued monitoring remains essential to address long-term environmental effects and public concerns. Furthermore, as a country that has experienced a large-scale nuclear event, Japan’s expertise and long-term data can contribute meaningfully to the development of international guidelines and preparedness strategies for radiological food safety. Japan’s integrated approach provides a robust evidence-based model for enhancing global resilience to future radiological emergencies.
